# *SCYL1* variants cause a syndrome with low γ-glutamyl-transferase cholestasis, acute liver failure, and neurodegeneration (CALFAN)

**DOI:** 10.1038/gim.2017.260

**Published:** 2018-02-08

**Authors:** Dominic Lenz, Patricia McClean, Aydan Kansu, Penelope E Bonnen, Giusy Ranucci, Christian Thiel, Beate K Straub, Inga Harting, Bader Alhaddad, Bianca Dimitrov, Urania Kotzaeridou, Daniel Wenning, Raffaele Iorio, Ryan W Himes, Zarife Kuloğlu, Emma L Blakely, Robert W Taylor, Thomas Meitinger, Stefan Kölker, Holger Prokisch, Georg F Hoffmann, Tobias B Haack, Christian Staufner

**Affiliations:** 1 Division of Neuropediatrics and Pediatric Metabolic Medicine, Department of General Pediatrics, University Hospital Heidelberg, Heidelberg, Germany; 2 Children’s Liver Unit, Leeds Children’s Hospital, Leeds, UK; 3 Division of Pediatric Gastroenterology, Department of Pediatrics, Ankara University School of Medicine, Ankara, Turkey; 4 Department of Molecular and Human Genetics, Baylor College of Medicine, Houston, Texas USA; 5 Department of Translational Medical Sciences, Section of Pediatrics, Liver Unit, University of Naples Federico II, Italy, Italy; 6 Division of Metabolism, IRCCS Bambino Gesù Children’s Hospital, Rome, Italy; 7 Institutes of Pathology, University Hospital Heidelberg, Heidelberg, Germany; 8 Institute of Pathology and Tissue Bank of the, University Medical Center Mainz, Mainz, Germany; 9 Department of Neuroradiology, University Hospital Heidelberg, Heidelberg, Germany; 10 Institute of Human Genetics, Technische Universität München, Munich, Germany; 11 Department of Pediatrics, Section of Gastroenterology, Hepatology, and Nutrition, Texas Children’s Hospital, Houston, Texas USA; 12 Wellcome Centre for Mitochondrial Research, Institute of Neuroscience, The Medical School, Newcastle University, Newcastle upon Tyne, UK; 13 Institute of Human Genetics, Helmholtz Zentrum München, Neuherberg, Germany; 14 Institute of Medical Genetics and Applied Genomics, University of Tübingen, Tübingen, Germany

**Keywords:** acute liver failure, CALFAN syndrome, congenital disorder of intracellular trafficking, low-GGT cholestasis, *SCYL1*

## Abstract

**Purpose:**

Biallelic mutations in *SCYL1* were recently identified as causing a syndromal disorder characterized by peripheral neuropathy, cerebellar atrophy, ataxia, and recurrent episodes of liver failure. The occurrence of SCYL1 deficiency among patients with previously undetermined infantile cholestasis or acute liver failure has not been studied; furthermore, little is known regarding the hepatic phenotype.

**Methods:**

We aimed to identify patients with *SCYL1* variants within an exome-sequencing study of individuals with infantile cholestasis or acute liver failure of unknown etiology. Deep clinical and biochemical phenotyping plus analysis of liver biopsies and functional studies on fibroblasts were performed.

**Results:**

Seven patients from five families with biallelic *SCYL1* variants were identified. The main clinical phenotype was recurrent low γ-glutamyl-transferase (GGT) cholestasis or acute liver failure with onset in infancy and a variable neurological phenotype of later onset (CALFAN syndrome). Liver crises were triggered by febrile infections and were transient, but fibrosis developed. Functional studies emphasize that SCYL1 deficiency is linked to impaired intracellular trafficking.

**Conclusion:**

SCYL1 deficiency can cause recurrent low-GGT cholestatic liver dysfunction in conjunction with a variable neurological phenotype. Like NBAS deficiency, it is a member of the emerging group of congenital disorders of intracellular trafficking causing hepatopathy.

## Introduction

Acute liver failure (ALF) in infancy is a rare but life-threatening event.^1^ In Europe, pediatric ALF is mostly caused by infections and inherited metabolic diseases; however, in approximately 50% the etiology remains unresolved.^1,2^ Meanwhile, infantile cholestasis is a relatively common clinical condition affecting 1 in 2,500 children,^3^ mainly caused by biliary atresia, infections, and inherited metabolic diseases. Infantile cholestasis with normal or low serum γ-glutamyl-transferase (GGT) activity is mostly caused by genetic disorders, but in analogy to pediatric ALF, many patients remain undiagnosed.^4,5^ Unknown diagnosis hampers decision-making on appropriate treatment strategies. It has been speculated that a significant number of individuals with indeterminate pediatric ALF or infantile cholestasis suffer from as yet unknown inherited metabolic diseases.^4,6^

In recent years, whole-exome sequencing studies have revealed several “new” genetic disorders with pediatric liver disease such as defects in cytosolic aminoacyl transfer RNA (tRNA) synthetases,^7,8,9,10^ disorders of Golgi homeostasis,^11,12^ and congenital disorders of intracellular trafficking.^13^ Among these, biallelic mutations in *NBAS* (neuroblastoma amplified sequence) cause a clinical syndrome with a wide phenotypic spectrum ranging from isolated hepatopathy to a multisystemic disease with skeletal dysplasia, short stature, and absent to mild neurological abnormalities.^14,15^ NBAS is part of the syntaxin 18 complex, a soluble *N*-ethylmaleimide-sensitive-factor attachment receptor involved in the coat protein complex I (COPI) retrograde trafficking at the endosplasmic reticulum (ER) membrane.^13,15,16^

Recently, compound heterozygous mutations in *SCYL1* were shown to be associated with a syndrome characterized by peripheral neuropathy, cerebellar atrophy, ataxia, and recurrent episodes of ALF in three individuals from two families.^17^ Like NBAS, SCYL1 is involved in retrograde transport, scaffolding class II adenosine diphosphate ribosylation factors that couple COPI coat proteins to membranes for the formation of coatomer complexes;^18^ in possible association with this, SCYL1 is known to regulate Golgi morphology.^17,19^ However, specific disease mechanisms are yet to be elucidated.

Here, we report on seven individuals from five families identified with previously unreported mutations in *SCYL1,* expanding the clinical and genetic spectrum. SCYL1 deficiency may lead to a predominant cholestatic phenotype with variable neurological features, which we suggest to name CALFAN syndrome (low γ-glutamyl-transferase cholestasis, acute liver failure, and neurodegeneration). *In vitro* studies on patients’ fibroblasts contribute to understanding disease mechanisms through analyses of ER stress, retrograde transport, and glycosylation.

## Materials and methods

### Patients

Seven individuals from five families with biallelic *SCYL1* variants, identified via a whole-exome sequencing study of patients with infantile cholestasis or ALF of unknown etiology, were studied in detail by a prospective observational follow-up study and by thoroughly evaluating the medical history. All procedures followed were in accordance with the ethical standards of the responsible committee on human experimentation (institutional and national) and with the Helsinki Declaration of 1975, as revised in 2000. Informed consent to participate in the study was obtained from all patients or their parents in case of minor patients. Additional informed consent was obtained from all patients for whom identifying information is included in this article. The study was approved by the ethical committee of the Technische Universität München and the ethical committee of the University Hospital Heidelberg.

### Exome sequencing and variant filtering

Subjects F1:II.2, F2:II.5, F2:II.6, F4:II.1, F4:II.2, and F5:II.3 were sequenced in Munich by whole-exome sequencing using the SureSelect Human All Exon V5 or V6 kit (Agilent, Santa Clara, CA) followed by sequencing as 100-bp paired-end runs on an Illumina HiSeq2500 or HiSeq4000 (Ilumina, San Diego, CA).^20^ After alignment to the human reference genome (University of California–Santa Cruz Genome Browser build hg19 http://hgdownload.cse.ucsc.edu/downloads.html) using Burrows–Wheeler Aligner (v.0.7.5a https://sourceforge.net/projects/bio-bwa/)^21^ single-nucleotide variants and small insertions and deletions (indels) were detected with SAMtools (version 0.1.19 http://samtools.sourceforge.net).^22^ Then, 8.1–11.8 Gb of sequences were mapped to the reference genome corresponding to a 102–140-fold coverage, with more than 97% of the target region being covered at least 20-fold (Supplementary Table S1 online). We applied different filtering steps to prioritize likely pathogenic variants including a phenotype-based search for rare (minor allele frequency <0.1% in in-house and public databases) recessive-type variants affecting genes that are listed in OMIM (phenotype key 3) and that have been associated with the search term *liver*. In all individuals, this search prioritized homozygous variants in a single gene, *SCYL1*. Subject F3.II.X received whole-exome sequencing in the clinical diagnostic laboratory at Baylor Genetics Laboratories. Sequencing methodology was previously described and can be briefly summarized as Illumina (Ilumina, San Diego, CA) paired-end sequencing of a capture-enriched 42-MB target to an average 100 × coverage, with at least 95% of targeted bases having a minimum of 20 reads.^23^ Analysis of variants followed the guidelines of the American College of Medical Genetics and Genomics.^24^ DNA sequences of the identified *SCYL1* variants were deposited at ClinVar with the following accession numbers: NM_020680.3:c.1882C>T: SCV000611620; NM_020680.3:c.1433A>G: SCV000611621; NM_020680.3:c.256G>T: SCV000611622; NM_020680.3:c.169C>T: SCV000611623; NM_020680.3:c.314C>T: SCV000611624.

### Microscopy of fibroblasts and liver

For hematoxylin and eosin staining as well as immunohistochemistry, liver biopsies were routinely fixed in formaldehyde and embedded in paraffin. Immunohistochemistry and immunofluorescence microscopy were performed as previously described.^25,26^ For ultrastructural analysis, biopsies were fixed in glutaraldehyde and embedded in epon or processed from paraffin blocks to epon. Thin sections were analyzed in a transmission electron microscope (JEM 1400, JEOL, Freising, Germany).

Mouse monoclonal antibodies were against the lipid droplet-associated protein perilipin 2 (AP 125, Progen Heidelberg, Germany) and cytokeratin 7 (Clone OV-TL 12/30, Dako, Agilent Technologies, Santa Clara, CA); additionally, rabbit antisera were used against SCYL1 (Atlas Antibodies N3C2, Biozol, Eching, Germany). Secondary anti-mouse and anti-rabbit hrp-coupled antibodies were from Cell Signaling (Danvers, MA).

### *Ex vivo* studies in patient and control fibroblasts

Fibroblasts were collected from the included individuals after informed consent was obtained. A fibroblast control cell line was purchased from Merck (SCC058, Darmstadt, Germany). All fibroblast cell lines were tested for mycoplasma contamination.

### Western blot

Patient and control fibroblasts were cultivated in Dulbecco’s modified Eagle’s medium supplemented with 10% fetal bovine serum and 1% penicillin/streptomycin at 37 °C and 5% CO_2_. For western blots, cells were collected, washed in phosphate buffered saline with tween (PBST) (0.1% tween), and resolved in radioimmunoprecipitation assay buffer. Next, 50 μg of protein of every sample were separated on a 10% polyacrylamide gel. Primary antibodies against SCYL1 (Sigma-Aldrich, Munich, Germany; rabbit anti-human; dilution 1:1,000 in PBST (0.1% tween)), GRP78/BiP (Sigma-Aldrich, rabbit anti-human; dilution 1:1,000 in PBST (0.1% tween)), and β-actin (Sigma-Aldrich, mouse anti-human; dilution 1:10,000 in PBST (0.1% tween)), were incubated overnight. Secondary hrp-coupled antibodies (goat anti-rabbit or rabbit anti-mouse) were from Dianova (Hamburg, Germany) and used in a dilution of 1:10,000 in PBST (0.1% tween). Enhanced chemiluminescence of proteins was detected using a Vilberscan Fusion FX7 (Vilber Lourmat, Marne-la-Vallée, France). Protein levels were quantified using the software Bio-1D (Vilber Lourmat, Marne-la-Vallée, France).

### Isoelectric focusing of serum transferrin and serum ApoCIII

Isoelectric focusing of serum transferrin and ApoCIII was performed as described previously.^27,28^

### Brefeldin A assay immunofluorescence

Brefeldin A (BFA) assay (with concentration of 2.5 μg/ml BFA) to investigate the retrograde transport was performed as described previously.^29^

### ER stress analyses

ER stress was investigated using the ER stress Antibody Sampler Kit (Cell Signaling, Danvers, MA). Fibroblasts grown under normal cell culture conditions as described above were used and additionally, cells stressed by glucose starvation were studied. To starve the cells, Dulbecco’s modified Eagle’s medium (25 mM glucose) was removed; the fibroblasts were washed with phosphate buffered saline and then incubated for 12 h in Dulbecco’s modified Eagle’s medium with glucose concentration reduced to 0.5 mM glucose, 10% fetal bovine serum, and 1% penicillin/streptomycin at 37 °C and 5% CO_2_.

## Results

**Figure 1:**
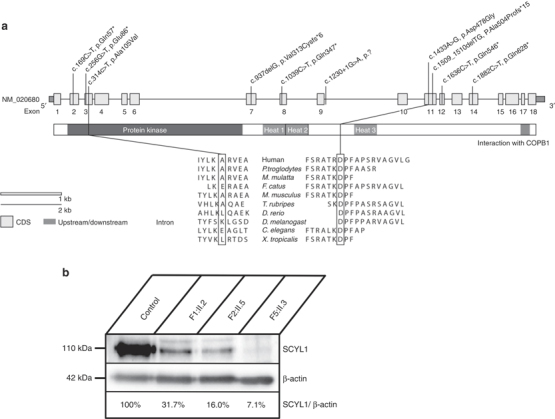
Genetic structure of *SCYL1* and proof of pathogenicity in case of missense variants. (**a**) Genetic structure of *SCYL1* including novel and previously reported mutations. (**b**). Biallelic *SCYL1* mutations lead to reduced protein levels of SCYL1; western blot for SCYL1. CDS, coding DNA sequence.

Seven patients from five families with novel biallelic mutations in *SCYL1* were detected (Table 1 and Figure 1a). Two individuals from a Pakistani family and the Italian individual are homozygous for a missense mutation, whereas the other individuals (descending from German and Turkish parents) are homozygous for nonsense mutations in *SCYL1*.

**Table 1:** Genetic and clinical phenotype of patients with CALFAN syndrome

**ID**	**Sex**	**Descent**	***SCYL1*** **variants**	**Phenotypic features**									
			**cDNA (NM_020680.3) protein (NP_065731.3)**	**Age at last examination**	**No. of episodes of liver dysfunction (age at first and last episode)**	**Trigger of liver dysfunction**	**Hepatomegaly**	**Liver biopsy (age at liver biopsy)**	**Cognitive function**	**Motor function**	**Microcephaly**	**cMRI abnormalities (age at MRI)**	**Other pathological findings**
F1:II.2	M	German	c.[1882C>T]; [1882C>T], p.[Gln628*]; [Gln628*]	3 y	4 (11 m to 1 y 10 m)	Febrile infections	Yes, during crisis	Lipid droplet inclusions portal and periportal fibrosis with ductular reactions (15 m)	Speech development delay	Transient tumbling, proximal muscle weakness	Yes, secondary (-2.94 SDS)	Unspecific T2-hyperintense point-shaped lesions in the subcortical white matter (1 y 11 m)	Uretero–pelvic junction obstruction
F2:II.5	F	Pakistani	c.[1433A>G]; [1433A>G], p.[Asp478Gly]; [Asp478Gly]	7 y	4 (7 m to 2 y 6 m)	Febrile infections	Yes, during crisis	Mild hepatic fibrosis and ductular reaction, microvesicular steatosis (11 m)	Lower end of the learning spectrum of the class	Normal	Yes, secondary (-2.44 SDS)	ND	None
F2:II.6	M	Pakistani	c.[1433A>G]; [1433A>G], p.[Asp478Gly]; [Asp478Gly]	3 y	4 (5 m to 2 y 5 m)	Febrile infections	Yes, during crisis, plus splenomegaly	Posthepatitic pattern with some features of resolving giant cell hepatitis, no evidence of fatty change (6 m)	Speech development delay	Normal	Yes, secondary (-3.89 SDS)	ND	Short stature, failure to thrive
F3:II.4	F	German	c.[256G>T]; [256G>T], p.[Glu86*]; [Glu86*]	10 y	4 (6 m to 21 m), age at transplant: 23 m	Febrile infections	Yes, plus splenomegaly	Pan lobular cholestasis with hepatocyte degeneration and focal giant cell transformation (6 m) stage 3–4 bridging fibrosis and nodularity suggesting cirrhosis, extensive hepatocellular injury, mild lobular inflammation, nonspecific cholangitis (13 m), stage 3–4 bridging fibrosis with focal nodularity, and rare focal chronic portal inflammation (23 m, at time of transplant)	Speech development delay, intellectual borderline deficiency (WASI-II FSIQ = 78)	Delayed, frequent falls, mild proximal weakness, mild action tremor (possibly secondary to tacrolimus)	Yes (-3.28 SDS)	No pathological findings (2 y and 9 y)	Intrauterine growth retardations, short stature, failure to thrive, hip dysplasia, coronal clefting of ribs, scoliosis
F4:II.1	F	Turkish	c.[169C>T]; c.[169C>T], p.[Gln57*]; [Gln57*]	11 y 10 m	3 (4 y to 6 y 11 m)	Febrile infections	Yes, progressive, plus splenomegaly	Bridging necrosis and fibrosis, moderate portal and lobular inflammation (4 y 1 m)	Speech development delay, mild mental retardation (IQ 50–69), stuttering	Proximal muscle weakness, wide-based gait, Gowers’ sign, tremor in hands	Yes (-2.55 SDS)	Mild cerebral and cerebellar atrophy, right frontal perivascular gliotic focus (10 y)	Nephrotic syndrome, lumbar lordosis
F4:II.2	F	Turkish	c.[169C>T]; c.[169C>T], p.[Gln57*]; [Gln57*]	8 y 8 m	5 (10 m to 8 y 6 m)	Febrile infections	Yes, progressive, plus splenomegaly	Portal fibrosis, porto-portal bridging, perisinusoidal, pericellular fibrosis (3 y 10 m)	Speech development delay, mild mental retardation (IQ 50–69), stuttering	Frequent falling, proximal muscle weakness, tremor in hands, step inwardly, steppage gait	No (-1.55 SDS)	Mild cerebral and cerebellar atrophy, venous anomaly at right basal ganglia (7 y)	Epilepsy, coarse face with prominent ala nasi, hemangioma on forehead, lumbar lordosis, palmar and plantar hyperkeratosis, hyperelasticity, pubertas precox
F5:II.3	M	Italian	c.[314C>T]; c.[314C>], p.[Ala105Val]; [Ala105Val]	4 y 9 m	3 (18 m to 4 y 5 m)	Febrile infections	Yes, during crisis	Acute injury with ductular proliferation (18 m)	Speech development regression, severe ID (GMDS-ER <1 percentile)	Motor stereotypies	Yes (-2.42 SDS)	T2 and FLAIR-hyperintensity of the subcortical white matter, slight dilatation of perivascular spaces (3 y 2 m)	Bilateral cryptorchidism, scrotal hernia

CALFAN, cholestasis or acute liver failure with onset in infancy and a variable neurological phenotype of later onset; cDNA, complementary DNA; cMRI, cerebral magnetic resonance imaging; F, female; FLAIR, fluid-attenuated inversion recovery; FSIQ, full scale intelligence quotient; GMDS-ER, Griffiths Mental Development Scales, extended revised; ID, intellectual disability; IQ, intelligence quotient, M, male; MRI, magnetic resonance imaging; ND, not determined; SDS, standard deviation score; WASI, Wechsler Abbreviated Scale of Intelligence.

### Exemplary case report of individual F1:II.2 and clinical phenotype of identified individuals with *SCYL1* variants

The first individual identified was F1:II.2, a boy, born to healthy, nonconsanguineous German parents at term with normal birth weight (for anthropometric data see Supplementary Table S2). He had neonatal hyperbilirubinemia that resolved without phototherapy. At the age of 11 months he presented with scleral jaundice 1 week after a febrile illness with diarrhea and fatigue. The laboratory workup showed a cholestatic pattern with direct hyperbilirubinemia, clearly increased aspartate aminotransferase (ASAT), mildly impaired liver function, and only marginally elevated GGT (Table 2). Laboratory findings resolved within a month but ASAT activity remained slightly elevated at levels around 60 U/L. Three similar episodes associated with febrile infections occurred until the age of 1 year 11 months. Hepatomegaly was present only during crises. The metabolic workup revealed pathological glycosylation patterns during liver crisis at the age of 1 year 11 months (Supplementary Figure S1). However, further samples did not show this finding. Other metabolic testing, including analysis of urinary bile acids, and investigation for infectious and immunological causes were negative. Repeated abdominal ultrasounds were unremarkable, but an abdominal magnetic resonance image (MRI) at the age of 1 year 11 months showed inhomogeneous perfusion suggesting liver fibrosis. Despite additional intercurrent febrile illnesses, no more bouts of liver dysfunction occurred after the age of 1 year 11 months.

**Table 2:** Laboratory findings during liver crises

**Patient**	**Number of liver crises (age at first–last crisis)**	**Max. ALAT (U/L) min–max (median)**	**Max. ASAT (U/L) min–max (median)**	**Max. total bilirubin (μmol/L) min–max (median)**	**Max. direct bilirubin (μmol/L) min–max (median)**	**Max. GGT (U/L) min–max (median)**	**Max. AP (U/L) min–max (median)**	**Max. INR min–max (median)**	**Min. albumin (g/L) min–max (median)**
**F1:II.2**	4 (11 m–1 y 11m)	79–880 (183)	230–1,964 (732)	17–118 (106)	12–102 (90)	13–77 (69)	403–1,197 (938)	1.08–1.56 (1.55)	33.9–39 (36)
**F2:II.5**	4 (7m–2 y 7 m)	537–1,113 (750)	ND	3–234 (119)	180^a^	30–105 (83)	1,378–2,533 (2,113)	1.3–3.0 (1.85)	30–41 (40.5)
**F2:II.6**	4 (5 m–2 y 5 m)	621–1,689 (1,090)	ND	12–379 (42)	239^a^	23–37 (34)	284–1,821 (501)	0.9–5.0 (1.25)	36–42 (41)
**F3:II.4**	4 (6 m–1 y 9 m)	481–1,387 (699)	979–1,526 (1,326)	65–325 (130)	51–79 (65)	38–97 (63)	1,071–1,526 (1,286)	1.9–4.2 (3.05)	34–44 (39)
**F4:II.1**	3 (4 y–6 y 11 m)	237–2,453 (497)	666–4,804 (1,424)	207–438 (335)	11–381 (186)	34–94 (39)	468–915 (889)	2.21–2.78(2.49)	26–33 (30)
**F4:II.2**	5 (10 m–8 y 6 m)	906–2,131 (1,024)	997–4,034 (1,784)	86–341 (209)	50–172 (155)	37–102 (60)	389–1,640 (549)	1.62–3.28 (2.93)	26–36 (30)
**F5:II.3**	3 (18 m–4 y 5 m)	59–770 (324)	61–1,780 (821)	7–410 (157)	15–310 (130)	31–55 (42)	218–822 (615)	1.12–1.65 (1.48)	34.9–44.1 (39.2)
Reference range		<50	<43	<17	<5	<60	96–311	<1.2	36–50

ALAT, alanine aminotransferase; AP, alkaline phosphatase; ASAT, aspartate aminotransferase; GGT, γ-glutamyl-transferase; INR, international normalized ratio; ND, not determined PT, prothrombin time.

^a^Measured once only.

At the age of 1 year 9 months, the boy was thoroughly examined by a pediatric neurologist, focusing on neurological abnormalities as observed in the first SCYL1 patients described by Schmidt et al.^17^ Apart from secondary microcephaly, no clinical abnormalities were noted. The family reported slightly delayed speech development and transient stuttering. Psychomotor development was normal, and there was no loss of neurological capabilities. Cognitive testing resulted slightly below average (Supplementary Table S3).

At the age of 3 years 1 month, the parents reported frequent falling. Electroencephalography remained unremarkable, cerebral MRI revealed nonspecific T2-hyperintensity of the subcortical frontal, parietal, temporal, and subinsular white matter, consistent with incomplete myelination, as well as punctate frontal and parietal temporal fluid attenuation inversion recovery hyperintensities, consistent with small areas of gliosis. Neither cerebellum nor optic nerves were atrophic, cerebrospinal fluid spaces were normal, and diffusion was not restricted (Supplementary Figure S2). MRI of the spine was normal. The falls were most likely due to a minor weakness of the torso and the proximal lower extremities. At the last examination at the age of 3 years 7 months, all signs had disappeared and the boy was asymptomatic (for gait analysis, see Supplementary Video S1).

In the further course of the study, we identified six other individuals from four families with biallelic mutations in *SCYL1* (Table 1; for detailed case reports see Supplementary Case Reports). Three of seven suffered from neonatal jaundice, some requiring phototherapy. All seven individuals presented with a severe cholestatic liver crisis triggered by a common febrile illness in their first 18 months of life, all following a similar pattern: laboratory workup showed increased alanine aminotransferase (ALAT), mildly impaired liver function, and only marginally elevated GGT (Table 2). Hepatomegaly was present in all children during crisis; four showed an additional splenomegaly. Metabolic, immunological, and microbiological testing did not reveal an underlying disease cause. No glycosylation abnormalities were observed apart from the transient findings during liver crisis in individual F1.II:2. Apart from ASAT activity, which remained slightly elevated in two patients, laboratory findings resolved within weeks, whereas liver fibrosis developed in all patients (Table 1). Three to five similar episodes occurred until early school age; only one individual still experiences ongoing crises with 8 years (F4:II.2). Individual F3:II.4 received liver transplantation at 23 months of age.

Six patients are microcephalic; six patients showed a mild language delay. Three individuals have a borderline to mild mental retardation and one individual suffers from severe intellectual disability. Motor dysfunction was present in five individuals with a very variable phenotype including mild proximal muscle weakness (four patients), tremor in their hands (three patients), and a remarkable gait (two patients; see Supplementary Video S2). Individual F4:II.2 suffered from seizures necessitating transient pharmaceutical therapy (for an overview of neurological findings, see Supplementary Table S3).

Skeletal abnormalities were present in five patients including short stature (F2:II.6, F3:II.4); hip dysplasia, coronal clefting of ribs, scoliosis (F3:II.4); and a coarse face, prominent ala nasi, heyperelasticity, and lumbar lordosis (F4:II.2). Lumbar lordosis was also present in F4:II.1, and F5:II.3 showed one lumbar vertebra with a “nail shot” appearance.

MRI findings showed signs of mild cerebral and cerebellar atrophy in two patients (F4:II.1, F4:II.2). No cerebellar vermis atrophy or optical nerve thinning was observed. Further clinical information is listed in Table 1 and Supplementary Table S3.

### Morphological characterization of hepatocytes/liver biopsy

**Figure 2:**
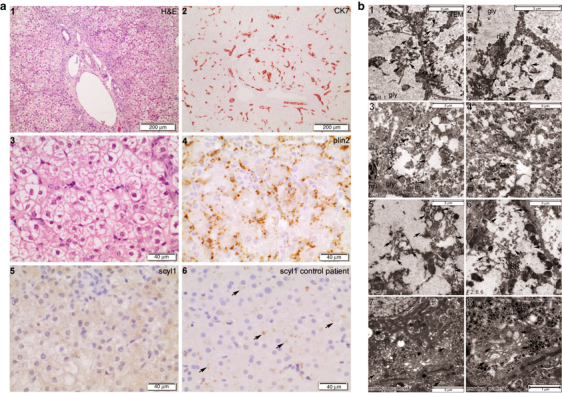
SCYL1 deficiency causes microvesicular steatosis, fibrosis, and disorganized Golgi apparatus. (**a**) Hematoxylin and eosin (H&E) stained liver biopsy shows hepatocytes with light-colored cytoplasm, incomplete cirrhosis (1, 3), CK7-positive ductular proliferations (2), and mild microvesicular steatosis as demonstrated by immunostains against plin2 (4). Antibodies against scyl1 stain diffusely the cytoplasm (5), but in contrast to control patients, no dot-like staining pattern is observed (6). (**b**) In transmission electron microscopy, enlarged Golgi cisternae are detected (1–6, arrows delineate margins). (7) and (8) show normal Golgi apparatus in a control patient. bc, bile canaliculus; gly, glycogen; rER, rough endoplasmic reticulum.

**Figure 3:**
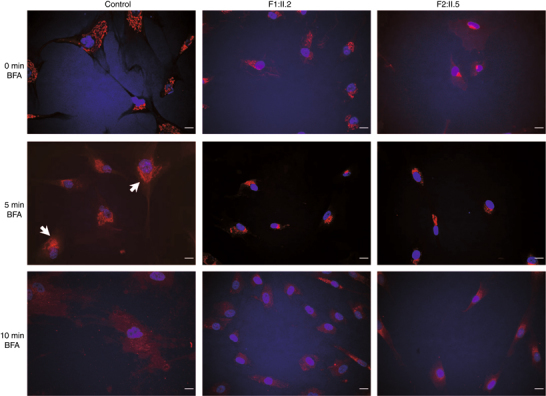
Brefeldin A (BFA) assay showing delayed retrograde transport. Control and patients’ fibroblasts (F1:II.2 and F2:II.5) were incubated for 0–10 min with BFA. Cells were fixed and analyzed by immunofluorescence for localization of Golgi marker GM130 (red). The cell nucleus (blue) was stained with DAPI. The white arrows indicate signal degradation of GM130 already after 5 min BFA treatment in the control cells in contrast to the patients’ fibroblasts. Bar: 10 μm.

In all seven individuals one or more liver biopsies (see Table 1, Figure 2) were performed. The most prominent finding was microvesicular steatosis and fibrosis (Figure 2a, 1,3,4). Positive cytokeratin 7 staining highlighted ductular proliferations as a sign of cholestasis (Figure 2a, 2). However, the biopsy of F2:II.6 showed a posthepatitic pattern with some features of resolving giant cell hepatitis and no evidence of fatty change. Focal giant cell formation was also seen in the first biopsy of F3:II.4. Later on, the explanted liver of that patient showed fibrosis with focal nodularity suggesting beginning cirrhosis. Antibody staining against SCYL1 showed depletion of SCYL1 in the patients’ tissue (Figure 2a, 5) compared with controls (Figure 2a, 6). When transmission electron microscopy images of hepatocytes were searched for Golgi apparatuses, first, no intact Golgi was found. Instead, in areas near bile canaliculi, greatly enlarged vesicular cisternae were observed, which correlated to extremely disorganized and hardly recognizable Golgi structures (see arrows in Figure 2b, 1–6; in comparison normal Golgi apparatuses are shown in a control patient with mitochondriopathy, Figure 2b, 7,8).

### Glycosylation studies

In the diagnostic workup during the episode of liver dysfunction at the age of 1 year 11 months in individual F1:II.2, isoelectric focusing of transferrin and apolipoprotein CIII in serum was performed followed by in-gel immunodetection. Pathological glycosylation patterns were observed during an episode of liver dysfunction, with profiles similar to a pattern typical for congenital disorder of glycosylation type II (CDG-II), indicating a generalized *N*-glycosylation and *O*-glycolysation (mucin-type) deficiency (Supplementary Figure S1a,b). However, further serum samples taken after this last episode of liver dysfunction revealed normal patterns for both marker proteins. Isoelectric focusing of serum transferrin in individuals F2:II.5 and F2:II.6 as well as in F5:II.3 was unremarkable (data not shown).

### Western blot

To investigate the expression level of SCLY1 in patients’ fibroblasts, western blot analysis was performed in fibroblasts of individuals F1:II.2, F2:II.5/F2:II.6, and F5.II.3. In contrast to the controls, expression of SCYL1 was severely reduced in patient cells, in the German patient homozygous for the nonsense mutation c.[1882C>T], p.[Gln628*], the Pakistani patient homozygous for the missense mutation c.[1433A>G], p.[Asp478Gly], and the Italian patient homozygous for the missense mutation c.[314C>T], p.[Ala105Val] (Figure 1b). Fibroblasts of F3.II.X, F4:II.1, and F4:II.2 were not available.

### Functional characterization of fibroblasts including BFA assay and ER stress analyses

The macrocyclic lactone BFA blocks anterograde traffic between the ER and the Golgi without affecting retrograde transport from the Golgi to the ER leading to a redistribution of preexisting *cis* and medial Golgi enzymes to the ER.^30^ Within minutes of BFA addition, tubular structures containing Golgi enzymes can be seen to move along microtubules from the Golgi to the ER in wild-type cells.^31^ After addition of BFA to patients’ fibroblasts, a delayed cellular dispersion of GM130-stained Golgi structures was observed when compared with controls pointing at an impaired delayed retrograde transport from the Golgi in SCYL1-deficient fibroblasts (Figure 3).

Western blot analysis for BIP/Grp78 as a possible downstream pathway of ER stress did not show significant alterations under normal or starving conditions (data not shown).

## Discussion

Here we report on seven individuals from five families with novel mutations in *SCYL1.* The variants caused either clear loss of function or—in the case of the two missense variants—were confirmed to be pathogenic through distinct loss of SCYL1 protein in patient tissues. Variants in *SCYL1* are described to cause spinocerebellar ataxia in an autosomal recessive manner (MIM 616719).^17^ This phenotype was predominantly neurological including optic atrophy, peripheral neuropathy, ataxia, tremor, and intellectual disability (Supplementary Table S3) whereas liver crises triggered by infections were mentioned without further detailed information.

The first and major clinical symptom in our patients with SCYL1 deficiency was recurrent low-GGT cholestatic liver dysfunction with developing fibrosis, while neurological signs and symptoms varied and had a later onset. We therefore suggest the acronym CALFAN (low-GGT cholestasis, acute liver failure, and neurodegeneration) syndrome for this clinical phenotype. Minor febrile infections triggered three to five episodes of liver crises, which did not always fulfill criteria of an ALF according to the criteria developed by the pediatric ALF study group.^1^ It remains unclear if the previously reported SCYL1-deficient individuals had ALF fulfilling these criteria, as laboratory findings or assessment of hepatic encephalopathy were not provided.^17^ A further, more recent case report by Smith et al.^32^ described another patient with SCYL1 deficiency with a pattern of recurrent hepatopathy—not mentioning liver failure. Apart from liver dysfunction, global developmental delay, poor growth, chronic anemia, skeletal dysplasia, abnormal MRI signal in the pons, an abnormal gait, and signs of a beginning peripheral neuropathy were reported in this patient.

Liver function in our patients recovered completely between episodes, while fibrotic tissue remodeling occurred in all seven patients. Crises started mainly after a minor febrile infection with a cholestatic liver dysfunction presenting with elevated ASAT and ALAT activities (ASAT > ALAT), increased (direct) bilirubin, and impaired coagulation, whereas GGT was typically normal or only mildly elevated, presenting low-GGT cholestasis (Table 2). In families I–III, first crises occurred at the age of 5–11 months (median 6.5 months), and there were no episodes of liver dysfunction noted after the age of 21–30 months (median 25.5 months) (Tables 1 and 2). However, the crises in individual F5:II.3 started at 18 months and he recently experienced the last crisis at 4 years 5 months. Furthermore, the siblings F4:II.1 and F4:II.2, carrying a homozygous stop mutation at the beginning of the gene, still experienced liver crises at school age (last crisis so far at the age of 8 years 6 months), while onset of liver dysfunction was 10 months in one and 4 years in the other sibling. Individual F3:II.4 received liver transplantation at the age of 23 months after having experienced a fourth crisis and a biopsy showing stage 3–4 bridging fibrosis and nodularity suggesting cirrhosis besides extensive hepatocellular injury. The outcome of the transplantation has been satisfactory until now (8 years post transplantation). She has suffered no further episodes of hepatic decompensation afterwards and is generally in good health. Concerning neurological aspects, secondary microcephaly was present in six individuals with mild speech delay also in five subjects, speech regression in one, and mild cognitive impairment in three (Supplementary Table S3). Individual F5:II.3 showed a severe intellectual disability, documented for the first time at the age of 20 months; furthermore he showed motor stereotypies. The two subjects who are homozygous for a stop mutation at the beginning of the gene (F4:II.1 and F4:II.2) displayed mild motor delay with frequent falls, mild proximal muscle weakness, and an abnormal gait (Table 1,Supplementary Table S3) with one individual showing hints of a mild polyneuropathy. However, in contrast to previously reported SCYL1 patients, no ataxia was observed in any subjects in this study. MRI of the brain was ascertained for individual F1:II.2 at the age of 2 years, for F3:II.4 at the ages of 2 and 9 years, and for F5:II.3 at the age of 3 years and no abnormalities/changes of brainstem and cerebellum were reported, whereas mild brain atrophy was reported at the age of 10 years in individual F4:II.1 and at the age of 7 years in individual F4:II.2.

The marked neurological phenotype and cranial MRI abnormalities of the previously published patients with *SCYL1* variants are in line with the Scyl1-deficient mouse, which has been known as mdf (muscle deficient) mouse since 1980.^17,32^ It is affected by progressive gait ataxia, cerebellar vermis atrophy, Purkinje cell loss, and optic nerve thinning.^33,34^ The previously published patients developed cerebellar dysfunction during early childhood. Onset of neurogenic stutter diverged between 3 and 20 years; the age of onset of peripheral neuropathy signs was not precisely reported.^17^ These more severe neurological signs were absent in the majority of the patients presented in this study at ages 11, 10, 8, 7, 3, or 3 years respectively, despite the fact that our patients harbor loss-of-function mutations, whereas patients F4:II.1, F4:II.2, and F5:II.3 partly resemble these features (see Supplementary Table S3 online for comparison of neurological phenotypes).

The phenotypic variability highlights that variants in *SCYL1* may cause primary or even isolated cholestatic hepatopathy in conjunction with a range of mild to more marked neurological phenotypes. The most explicit neurological findings were noticed in the patients with either early stop (F3 and F4) or early missense mutations (F5), which may point at a possible genotype–phenotype correlation. Interestingly, two subjects reported in this study have short stature and one has skeletal dysplasia, which has been previously associated with SCYL1 deficiency.^32^ These clinical features are reminiscent of NBAS deficiency delineating a further link between these two diseases affecting the retrograde intracellular transport.^14,15^ The phenotypic spectrum of SCYL1 deficiency will be further clarified by the identification of new patients and their detailed clinical characterization and follow-up. Liver transplantation seems a potentially successful therapeutic strategy for hepatic disease in CALFAN syndrome, but the advantageous disease course regarding the decreasing incidence and attenuation of liver dysfunction episodes with age in most patients will need to be considered when discussing liver transplantation as a therapeutic option.

The mechanism of brain and liver dysfunction in CALFAN syndrome remains unclear and open questions remain regarding the physiological role of SCYL1. As far as it is understood, SCYL1 plays a role in forming coatomer constructs for COPI vesicle traveling from the Golgi to the ER and is therefore involved in the retrograde transport.^18^ The results of the BFA assay underline a role of SCYL1 in retrograde transport (Figure 3). Furthermore, enlargement of the Golgi apparatus has been shown before in fibroblasts of SCYL1-deficient individuals.^17,19^ Transmission electron microscopy of liver biopsies in our patients demonstrates a dispersed rather than enlarged pattern of Golgi structures (Figure 2) as a possible link between Golgi morphology and transport.

ER stress is a possible common pathway associated with congenital disorders of intracellular trafficking.^13^ In our study no evidence for increased ER stress was found in normal or starving conditions of patients’ fibroblasts. It needs to be elucidated whether ER stress plays a pathomechanistic role in SCYL1-deficient hepatocytes. As elevated temperature is known to induce ER stress,^35^ early antipyretic treatment may be considered in patients with CALFAN syndrome.

In addition to its role in intracellular trafficking, SCYL1 is thought to be a part of the aminoacylation-dependent nuclear tRNA export machinery.^36^ This is of interest as cytosolic aminoacyl-tRNA synthetase deficiencies are associated with infantile hepatopathy and variable neurological phenotypes,^7,8,9^ linking SCYL1 deficiency and cytosolic aminoacyl-tRNA synthetase deficiencies. In individual F1:II.2, we observed pathological glycosylation of transferrin and apolipoprotein C during one crisis comparable with a CDG-II pattern. However, in patient F1:II.2 as well as in F2:II.5, F2:II.6, and F5:II.3 isoelectric focusing of serum transferrin was repeatedly normal in the interval, demonstrating that there is no permanent glycosylation defect in CALFAN syndrome. The observed CDG II pattern during liver crisis in patient F1:II.2 is likely to be secondary to liver dysfunction, possibly reflecting temporarily impaired Golgi function.

In conclusion, biallelic variants in *SCYL1* can cause recurrent infantile cholestasis with a variable neurological phenotype. Most notably, it should be considered as a differential diagnosis in low-GGT cholestasis. We recommend that children with history of episodic cholestatic liver dysfunction, with or without neurological symptoms, should undergo genetic testing of *SCYL1*. In analogy to NBAS deficiency, CALFAN syndrome contributes to the emerging group of congenital disorders of intracellular trafficking.

## Electronic supplementary material

Supplementary Information

Supplementary Information

Supplementary Table S1

Supplementary Table S2

Supplementary Table S3

Supplementary Video S1

Supplementary Video S2

Supplementary Figure S1

Supplementary Figure S2

## Electronic supplementary material

Supplementary material is linked to the online version of the paper at 10.1038/gim.2017.260
