# Supplementary material for: SCYL1 variants cause a syndrome with low γ-glutamyl-transferase cholestasis, acute liver failure, and neurodegeneration (CALFAN)
Source: Genet Med. 2018 Feb 8;20(10):1255–65. doi: 10.1038/gim.2017.260 (PMC5989927; doi:10.1038/gim.2017.260)
Supplement: Supplementary file 1 — Supplementary Information [file 41436_2018_205_MOESM1_ESM.docx]

**Case reports**

**Individual F1:II.2, a boy,** was born to healthy, non-consanguineous German parents at term with normal birth weight (for anthropometrical data see Table S2). He had neonatal hyperbilirubinemia which resolved without phototherapy. At the age of 11 months he presented with scleral jaundice one week after a febrile illness with diarrhea and fatigue. On physical examination hepatomegaly was present. The laboratory work-up showed a cholestatic pattern with direct hyperbilirubinemia, clearly increased ASAT, mildly impaired liver function and only marginally elevated GGT (Table 2). Laboratory findings resolved within a month but ASAT activity remained slightly elevated at levels around 60 U/L. Three similar episodes associated with febrile infections occurred until the age of 1 11/12 years. Hepatomegaly was only present during crises. The metabolic workup revealed pathological glycosylation patterns during liver crisis at the age of 1 11/12 years (Fig. 2 b/c). However, further samples did not show this finding. Other metabolic testing, including analysis of urinary bile acids, and investigation for infectious and immunological causes were negative. Repeated abdominal ultrasounds were unremarkable, but an abdominal magnetic resonance imaging (MRI) at the age of 1 11/12 years showed inhomogeneous perfusion suggesting liver fibrosis. Despite additional intercurrent febrile illnesses, no more bouts of liver dysfunction occurred after the age of 1 11/12 years.

At the age of 1 9/12 years, the boy was thoroughly examined by a pediatric neurologist, focusing on neurological abnormalities as observed in the first SCYL1 patients described by Schmidt et al 2015 ^[17](#_ENREF_17)^. Apart from secondary microcephaly, there were no clinical abnormalities noted. The family reported slightly delayed speech development and transient stuttering. Psychomotor development was normal, and there was no loss of neurological capabilities. Cognitive testing resulted was under the average (Table S3).

At the age of 3 1/12 years, the parents reported frequent falling. Electroencephalography remained unremarkable, cerebral MRI revealed non-specific T2-hyperintensity of the subcortical frontal, parietal, temporal, and subinsular white matter, consistent with incomplete myelination, as well as punctate frontal and parietal temporal fluid attenuation inversion recovery hyperintensities (Fig. S1), consistent with small areas of gliosis. Neither cerebellum nor optic nerves were atrophic, cerebrospinal fluid spaces were normal, and diffusion was not restricted (Fig. S1). MRI of the spine was normal. The falls were most likely due to a minor weakness of the torso and the proximal lower extremities. At the last examination at the age of 3 7/12 years, all signs had disappeared and the boy was asymptomatic.

The patients **F2:II.5, a girl, and F2:II.6, a boy,** were born to healthy consanguineous parents of Pakistani decent at term with normal birth weights (anthropometrical data see Table S2). They have four healthy siblings. Both patients had neonatal jaundice, the girl requiring three days of phototherapy. At the age of 7 months, she presented with jaundice 2 weeks after a febrile illness with cough and diarrhea. Four years later the younger brother had a 2 day history of fever and vomiting, followed by icterus, pale stools and dark urine at the age of 5 months as a first episode. On physical examination both had hepatomegaly but no splenomegaly. Laboratory testing showed increased ALAT and (direct) bilirubin as well as prolonged prothrombin time but only mildly elevated GGT (ASAT n.a., Table 2), which all normalized during follow-up. Until the age of 2 6/12 years, both individuals experienced three additional episodes, all triggered by febrile illnesses and with similar laboratory findings characterized by direct hyperbilirubinemia and elevated ALAT with low GGT and variable coagulopathy (Table 2). Metabolic, immunological and microbiological testing did not reveal an underlying disease cause. Both siblings presented hepatomegaly only during crises and hyperbilirubinemia, ALAT activities and liver function always normalized after each episode. After the fourth crisis at the age of 2 6/12 years, no further episodes occurred in both individuals in spite of several infections, including febrile diseases.

Concerning neurological characteristics, the younger sibling experienced mild language delay whilst the older sister was initially thought to be in the lower end of the learning spectrum but has caught up in the meantime; otherwise developmental progress was normal (Table S3). The younger brother suffered from impairment of expressive speech which resolved with therapy. At the last clinic visit at the age of 7 11/12 years and 3 9/12 years, respectively, neurological examinations of both were normal except for secondary microcephaly. They are smaller than their four healthy siblings and grow along the 2nd-4th centile for weight and height whereas their healthy siblings are near the 50^th^.

Patient **F3:II.4**, a female, was born to distantly related parents of German descent at full term (anthropometrical data see Table S2). She has two older half-siblings and one full sibling. Additionally, the mother had 3 consecutive miscarriages. Prenatal history was significant for short femurs and intrauterine growth restriction and in the first months of life she was noted to suffer from failure to thrive. A skeletal survey revealed only 11 sets of ribs with several thoracic and lumbar vertebrae that had coronal clefts. Additional skeletal findings included bilateral acetabulum with a flat horizontal appearance and bilateral hypoplastic iliac wings. At 6 months of age she was hospitalized following 5 days of jaundice associated with episodes of fever and diarrhea but no vomiting. ALAT, ASAT and bilirubin were markedly elevated while GGT was low (Table 2). Laboratory findings normalized during follow-up. The patient experienced a second hospitalization due to jaundice at age 9 months, further details of which were not available. At age 12 months she was admitted a third time to hospital due to another febrile illness followed by ALF with a cholestatic pattern and severely impaired function of hepatic synthesis (Table 2). At 21 months of age the individual presented to hospital with vomiting for 12 hours, diarrhea for 6 days and low-grade fever. Laboratory work-up again showed low GGT cholestasis. She received a liver transplant one month later. The explanted liver revealed bridging portal fibrosis (stage 3-4) with focal nodularity, and rare foci of chronic portal inflammation. The patient did not experience any additional hepatic crises post-transplant.

Language development was delayed. She had a history of repetitious speech and began speaking in sentences at age 7.5 years. Motor skills were delayed and the patient experienced frequent falls. Beginning at the age of 4 years, the patient was noted to have a mild action tremor of her hands, which appeared mostly static until the age of 8 years when it began to worsen. At age 9 years the girl began writing. Also at this time she was noted as having increased tremor, mild proximal muscle weakness, with normal muscle tone and bulk, and normal gait. Cognitive function was borderline low (Table S3). Additionally, she was noted as having poor social response and poor interest in others, but absent repetitive behaviors or restricted interests. Furthermore, she is microcephalic and of short statue (Table S2).

The patients **F4:II.1** and **F4:II.2**, both girls, were born to healthy, consanguineous Turkish parents at term by cesarean section – F4:II.1 with low, F4:II.2 with normal birth weight (Table S2). The older sister, **F4:II.1,** presented at the age of 4 years with fever, irritability, anorexia and fatigue. On physical examination hepatomegaly was present. The laboratory work-up showed increased ALAT and ASAT with mild direct hyperbilirubinemia and impaired liver function, while GGT was low. A few days later, full blown liver failure developed (Table 2). Clinical and laboratory findings resolved to normal within 45 days. In total, three episodes of hepatic dysfunction associated with febrile infections occurred until the age of 6 11/12 years, which were less severe than the first episode in terms of laboratory findings.

The younger sister, **F4:II.2,** underwent her first crisis at the age of 10 months after an episode of fever. She showed hepatomegaly and a similar laboratory pattern as her sister - also developing full blown liver failure in the further course. In total, she had five episodes of liver dysfunction associated with febrile infections until the age of 8 6/12 years. Compared to her elder sister, her attacks were more severe; in 2 episodes plasmapheresis was necessary.

In both siblings, extensive infectious, metabolic and immunological workup was normal. Lipid profile was checked and genetic analysis for Niemann-Pick type C was performed due to the presence of this disorder in a close relative, resulting unremarkable. Abdominal ultrasounds during follow-up revealed parenchymal heterogeneity and hyperechogenity in F4:II.2. Hepatomegaly persisted which gradually enlarged over years along with splenomegaly in both siblings. Despite further febrile episodes, the elder sister did have no more bouts of liver dysfunction after the age of 6 11/12 years (age at last examination 11 10/12 years), whereas the younger sister, aged 8 8/12 years at time of report, had a crisis only two months ago.

Growing up, the elder sister (F4:II.1) was diagnosed mild mental retardation with speech delay at the age of 6 years requiring special education. Furthermore stuttering, tremor in hands, lomber lordosis, wide-based gait and mild proximal muscle weakness were noticed (Table S3). A cranial MRI revealed a mild cerebral and cerebellar atrophy and a perivascular gliotic focus in the right frontal area.

In the younger sister (F4:II.2) mild facial dysmorphism including a coarse face and prominent ala nasi was noticed during the first hospitalisation at 10 months of age becoming more remarkable when she grew up. She developed convulsions at 2 8/12 years necessitating anti-epileptic therapy with levetiracetam being now free of seizures for more than 4 years. At 3 6/12 years a mild mental retardation with speech delay, requiring special education, was evident. She showed stuttering (at 4 years) and a tremor in hands (at 6 years). A palmar and plantar hyperkeratosis was present since birth. Lomber lordosis, hyperelasticity, inwardly stepping, steppage gait and mild proximal muscle weakness (Table S3) were noticed at 5 years of age. At this time, the patient presented frequent falling, resulting in bone fractures in separate occasions (humerus, tibia) although tramauta were mild. However, no clear signs of osteopenia or osteoporosis were reported. Puberta praecox was diagnosed at 7 6/12 years of age and luteinizing hormone releasing hormone analogue was started (still ongoing). Further endocrinological work-up showed no other pathologies. Cranial MRI showed no abnormal signals of the hypophysis but a mild cerebral and cerebellar atrophy and venous anomaly at right basal ganglia. At their last clinical visit aged 11 10/12 and 8 8/12 years, both sisters were in good health and laboratory values were within normal limits.

Individual **F5:II.3**, a boy, was born to healthy, non-consanguineous Italian parents at term by cesarean section for breech presentation (anthropometrical data see Table S2). He had a bilateral cryptorchidism and a scrotal hernia. At the age of 17 months he presented with scleral jaundice one week after a febrile illness with diarrhea, vomiting and fatigue. On physical examination hepatomegaly was present. The laboratory work-up showed a cholestatic pattern with severe direct hyperbilirubinemia, clearly increased ASAT and ALAT, persistent coagulopathy, only marginally elevated gamma glutamyl transferase (GGT) and low platelet count (Table 2). Laboratory findings resolved within 50 days but ASAT and ALAT activity remained slightly elevated at levels around 60 U/L. Bone marrow aspiration, carried out due to persistent thrombocytopenia, revealed an increased amount of histiocytes with several vacuoles in the cytoplasm and rare figures of phagocytosis. Liver biopsy revealed signs of acute injury with signs of regeneration (ductular proliferation) without inflammatory infiltrates. Two similar episodes occurred until the age of 4 5/12 years. Hepatomegaly was only present during crises. Metabolic, infectiological and immunological workup remained without specific findings. Repeated abdominal ultrasounds showed an inhomogeneous liver echogenicity suggesting fibrosis. The patient is microcephaly and has a long face. Furthermore, a cervical spine x-ray revealed a second lumbar vertebra with a “nail shot” appearance. From 2 years old he experienced a regression of speech associated with an autism spectrum disorder, characterized mainly by hyperactivity and tendency to isolation. An EEG remained unremarkable, cerebral MRI revealed non-specific T2 and fluid attenuation inversion recovery hyperintensities of the subcortical white matter, consistent with late myelination, as well as a slight dilatation of several Virchow-Robin perivascular spaces. Neither cerebellum nor optic nerves were atrophic and diffusion was not restricted. According to Griffiths Mental Development Scales-Extended Revised patient has at the last follow-up a mental age less than 24 months- < 1 percentile (with a score at sub-scale A at 20 percentile and at sub-scale B/C/D/E/F less than 1 percentile).

His brother received a diagnosis of Asperger syndrome at 8 years old; there is a familiarity of optic nerve drusen.
