# Supplementary material for: SCYL1 variants cause a syndrome with low γ-glutamyl-transferase cholestasis, acute liver failure, and neurodegeneration (CALFAN)
Source: Genet Med. 2018 Feb 8;20(10):1255–65. doi: 10.1038/gim.2017.260 (PMC5989927; doi:10.1038/gim.2017.260)
Supplement: Supplementary file 2 — Supplementary Information [file 41436_2018_205_MOESM2_ESM.docx]

Supplementary INFORMATION

**Case Reports. Detailed Case Reports for each study individual**

**Table S1. Details on sequencing of individuals F1:II.2, F2:II.5, F2:II.6, F4:II.1 and F4:II.2**

**Table S2. Anthropometric Data**

**Table S3. Neurological findings of individuals with biallelic *SCYL1* mutations**

**Figure S1.** **Glycosylation abnormalities in patient F1:II.2**

a) and b) Isoelectric focusing of serum-transferrin and ApoCIII in individual F1:II.2
*Abbreviations*: CDG: congenital disorder of glycosylation; COG: conserved oligomeric Golgi complex, m: months

Figure S2. MRI of individual F1:II.2 at 3.5 years

T2-hyperintensity of subcortical white matter (A, C-E), consistent with incomplete myelination, in combination with punctate T2/FLAIR-hyperintensities (B), consistent with non-specific gliosis. In contrast to the previously reported patient by Schmidt et al 2015 the cerebellum is normal (F, G). (T2w: A, C-E, FLAIR: B; T1w: F, G):

**Video S1.**  **Gait of individual F1:II.2**

**Video S2.**  **Gait of individual F4:II.1**
