# Supplementary material for: SCYL1 variants cause a syndrome with low γ-glutamyl-transferase cholestasis, acute liver failure, and neurodegeneration (CALFAN)
Source: Genet Med. 2018 Feb 8;20(10):1255–65. doi: 10.1038/gim.2017.260 (PMC5989927; doi:10.1038/gim.2017.260)
Supplement: Supplementary file 3 — Supplementary Table S1 [file 41436_2018_205_MOESM3_ESM.docx]

**Table S1. Details on sequencing of individuals F1:II.2, F2:II.5, F2:II.6, F4:II.1 and F4:II.2**

| **ID** | **Mapped** | **Mapped percent** | **Seq (Gb)** | **Read length** | **On bait** | **Avg cov (exome)** | **Uncovered** | **Cov 1x** | **Cov 4x** | **Cov 8x** | **Cov 20x** | **Ts/**  **Tv** | **SNV** | **Indel** | **Pindel** | **Exome depth** | **Insert (sd)** | **CNV noise (Rs)** |
| --- | --- | --- | --- | --- | --- | --- | --- | --- | --- | --- | --- | --- | --- | --- | --- | --- | --- | --- |
| **F1:II.2** | 104053228 | 99.4 | 10.57 | 101 | 78.66 | 130.65 | 0.07 | 99.93 | 99.72 | 99.35 | 97.41 | 2.92 | 65979 | 5370 | 650 | 157 | 201 (62) | 1.23 |
| **F2:II.5** | 79937759 | 99.32 | 8.13 | 101 | 80.26 | 102.36 | 0.07 | 99.93 | 99.73 | 99.34 | 96.99 | 2.91 | 66715 | 5640 | 320 | 96 | 203 (68) | 1.02 |
| **F2:II.6** | 117024651 | 99.82 | 11.84 | 101 | 75.83 | 140.45 | 0.23 | 99.77 | 99.59 | 99.36 | 97.87 | 2.98 | 67188 | 5786 | 314 | 155 | 214 (70) | 1.32 |
| **F4:II.1** | 156918329 | 99.91 | 15.850 | 101 | 77.04 | 167.51 | 0.29 | 99.71 | 99.56 | 99.40 | 98.60 | 3.02 | 78585 | 6621 | 793 | 212 | 203 (66) | 1.26 |
| **F4:II.2** | 132760747 | 99.11 | 13.410 | 101 | 75.67 | 140.10 | 0.22 | 99.78 | 99.61 | 99.41 | 98.24 | 3.03 | 78858 | 6600 | 753 | 198 | 204 (67) | 1.10 |
| **F5:II.3** | 111274463 | 99.88 | 11.240 | 101 | 76.38 | 116.89 | 0.10 | 99.90 | 99.79 | 99.60 | 98.32 | 3.08 | 79655 | 6446 | 751 | 174 | 215 (74) | 1.13 |
