# Supplementary material for: SCYL1 variants cause a syndrome with low γ-glutamyl-transferase cholestasis, acute liver failure, and neurodegeneration (CALFAN)
Source: Genet Med. 2018 Feb 8;20(10):1255–65. doi: 10.1038/gim.2017.260 (PMC5989927; doi:10.1038/gim.2017.260)
Supplement: Supplementary file 4 — Supplementary Table S2 [file 41436_2018_205_MOESM4_ESM.docx]

**Table S2. Anthropometric Data**

| **General** |  |  |  |  |  |  |  |
| --- | --- | --- | --- | --- | --- | --- | --- |
| ID | **F1:II.2** | **F2:II.5** | **F2:II.6** | **F3:II.4** | **F4:II.1** | **F4:II.2** | **F5:II.3** |
| Ethnicity | German | Pakistani | Pakistani | German | Turkish | Turkish | Italian |
| Sex | M | F | M | F | F | F | M |
|  |  |  |  |  |  |  |  |
| **Antropometric data at birth** |  |  |  |  |  |  |  |
| Gestational age | **Term** | **Term** | **Term** | **Term** | **Term** | **Term** | **Term** |
| Head circumference in cm (SDS) | 34.0 (-1.81) | 35.5 (-0,53)  at 3 weeks | 36.0 (-0.45)  at 17 days | n.a. | n.a. | n.a. | 32 (-2.24) |
| Length in cm (SDS) | 54.0 ( 1.26) | 66.0 (-1,31)  at 7 months | 52.0 (-1.07)  at 17 days | n.a. | n.a. | n.a. | 49 (-0.86) |
| Weight in g (SDS) | 3930 ( 1.42) | 3080 (-0.93) | 3550 (-0.17 ) | 2466 (-1.88) | 2280 (-2.27) | 2800 (-1.18) | 2550 (-1.90) |
|  |  |  |  |  |  |  |  |
| **Antropometric data at last visit** |  |  |  |  |  |  |  |
| Age at last visit in years | **3 7/12** | **7 11/12** | **3 9/12** | **9 3/12** | **11 10/12** | **8 8/12** | **4 9/12** |
| Head circumference in cm (SDS) | 48.3 (-2.55) | 50.0 (-2.44) | 46.4 (-3.89) | 49.4 (-3.28) | 51 (-2.55) | 50.6 (-1.55) | 49 (-2.42) |
| Height in cm (SDS) | 104.1 ( 0.40) | 121.1 (-1.91) | 92.5 (-2.87) | 117.4 (-2.82) | 135 (-1.94) | 129 (-0.36) | 105 (-0.63) |
| Weight in kg (SDS) | 15.1 (-0.38) | 22.4 (-1.04) | 12.1 (-2.35) | 23.6 (-1.44) | 39 (-0.07) | 27 (-0.19) | 16.8 (-0.63) |
| BMI (SDS) | 13.93 (-1.34) | 15.27 (-0.39) | 14.14 (-1.12) | 17.12 (0.29) | 21.30 (1.17) | 16.22 (-0.02) | 15.2 (-0.32) |

M, male; F, female; n.a., not available
