# Supplementary material for: SCYL1 variants cause a syndrome with low γ-glutamyl-transferase cholestasis, acute liver failure, and neurodegeneration (CALFAN)
Source: Genet Med. 2018 Feb 8;20(10):1255–65. doi: 10.1038/gim.2017.260 (PMC5989927; doi:10.1038/gim.2017.260)
Supplement: Supplementary file 5 — Supplementary Table S3 [file 41436_2018_205_MOESM5_ESM.docx]

**Table S3. Neurological findings of individuals with biallelic *SCYL1* mutations**

| **ID** | **F1:II.2** | **F2:II.5** | **F2:II.6** | **F3:II.4** | **F4:II.1** | **F4:II.2** | **F5:II.3** | **Schmidt et al 2015*** |
| --- | --- | --- | --- | --- | --- | --- | --- | --- |
| **Age at most recent clinical examination (years)** | 3 7/12 | 7 11/12 | 3 9/12 | 10 | 11 10/12 | 8 8/12 | 4 7/12 | 16-20 |
| **Walking independently (months)** | 15 | 14 | 15 | NA | 15 | 15 | 15 | 12-24 |
| **Cognition** | BAYLEY-II: 84 (norm 85-115) | generally good, struggling with mathematics | slight speech delay, but now OK | WASI-II FSIQ 78 (7th percentile) | mild ID (IQ 50-69) | mild ID (IQ 50-69) | severe ID GMSD-ER < 1 percentile | normal-mild ID |
| **Neurogenic stutter (age of onset, years)** | temporary | none | none | none | 10 years | 4 years | (2 years | +-++ (3-20) |
| **Cerebellar oculomotor disturbance** | none | none | none | none | none | none | none | + |
| **Limb ataxia (UL/LL)** | none | none | none | none | none | none | none | no - +/+ |
| **Gait ataxia (onset)** | none | none | none | none | wide-based gait  (9 years) | Inwardly stepping, steppage gait (5years) | none | + (early childhood – childhood) |
| **Muscle weakness** | mild proximal weakness | none | none | mild proximal weakness | mild proximal weakness,  Gowers sign + | mild proximal weakness | none | distal, LL>UL |
| **Muscle atrophy** | none | none | none | none | none | none | none | LL=UL |
| **Sensory deficits and symptoms** | none | none | none | none | none | none | decreased pain perception | decreased pain perception (distal LL) – intermittent distal paresthesias (UL and LL) |
| **Electroneurophysiology** | motoric: unremarkable; sensory: no cortical answers. | ND | ND | ND | none | PNP | ND | axonal PNP |
| **Stretch reflexes** | normal | normal | none | NA | normal | increased (UL) | normal | decreased (LL) – increased (UL>LL) |
| **Spasticity** | none | none | none | none | none | none (hyperelasticity+) | none | no- + (UL) |
| **Hip abductor weakness** | none | none | none | 4/5 at deltoids and hip flexors, 3/5 on foot dorsiflexion | none | none | none | no - + (LL, central pattern) |
| **Tremor** | none | none | none | mild action tremor | intention | intention | none | intention (cerebellar) - action |
| **Cerebellar vermis atrophy on MRI** | none | ND | ND | none | mild cerebellar atrophy | mild cerebellar atrophy | none | +-++ |
| **Optic nerve thinning on MRI** | none | ND | ND | none | none | none | none | + |
| **Other findings on MRI** | unspecific T2 hyperintense subcortical foci |  |  | none  (2 MRI at ages 2 yr & 9 yr, both normal) | mild cerebral and cerebellar atrophy;  right frontal perivasculer gliotic focus | mild cerebral atrophy and cerebellar atrophy and venous anomaly at right basal ganglia | T2 and FLAIR-hyperintensity of the subcortical white matter slight dilatation of perivascular spaces |  |
| **Microcephaly (SDS)** | yes, secondary SDS -2.55 | yes, secondary  SDS -2.44 | yes, secondary  SDS -3.89 | yes, secondary  SDS -3.28 | yes  (SDS -2.55) | no  (SDS -1.55) | yes,  (SDS -2.42) | ND |

ID, intellectual disability; UL, upper limbs; LL, lower limbs; PNP, peripheral neuropathy; NA, not available; ND, not determined; -, not present; +, present (mild);++, present (pronounced).

* Schmidt WM, Rutledge SL, Schule R, Mayerhofer B, Zuchner S, Boltshauser E, Bittner RE. Disruptive *SCYL1* Mutations Underlie a Syndrome Characterized by Recurrent Episodes of Liver Failure, Peripheral Neuropathy, Cerebellar Atrophy, and Ataxia. Am J Hum Genet 2015; 97:855-861.
