# Supplementary figures and images for: SCYL1 variants cause a syndrome with low γ-glutamyl-transferase cholestasis, acute liver failure, and neurodegeneration (CALFAN)
Source: Genet Med. 2018 Feb 8;20(10):1255–65. doi: 10.1038/gim.2017.260 (PMC5989927; doi:10.1038/gim.2017.260)

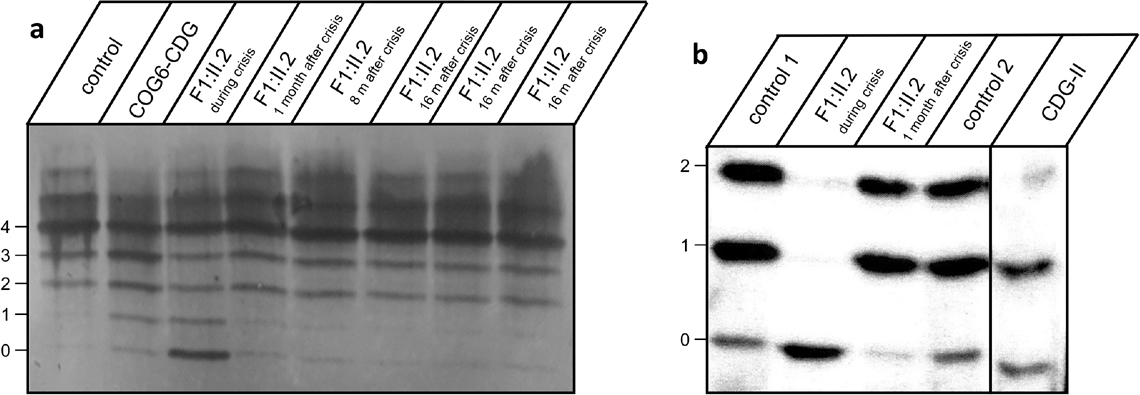

Supplement: Supplementary file 8 — Supplementary Figure S1 [file 41436_2018_205_MOESM8_ESM.jpg]

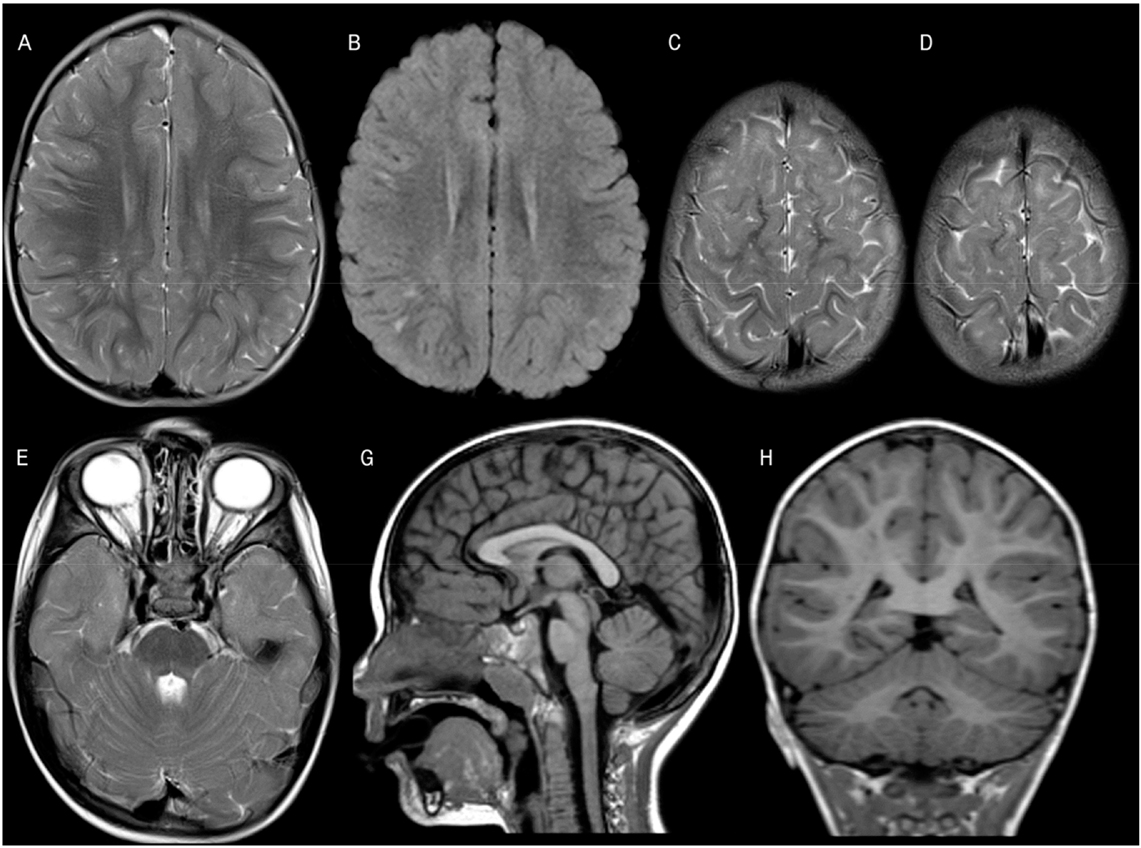

Supplement: Supplementary file 9 — Supplementary Figure S2 [file 41436_2018_205_MOESM9_ESM.jpg]
